# Virtual Therapy with the NMDA Antagonist Memantine in Hippocampal Models of Moderate to Severe Alzheimer’s Disease, In Silico Trials

**DOI:** 10.3390/ph15050546

**Published:** 2022-04-28

**Authors:** Dariusz Świetlik, Jacek Białowąs, Aida Kusiak, Marta Krasny

**Affiliations:** 1 Division of Biostatistics and Neural Networks, Medical University of Gdańsk, Dębinki 1, 80-211 Gdansk, Poland; 2 Division of Anatomy and Neurobiology, Medical University of Gdańsk, Dębinki 1, 80-211 Gdansk, Poland; jacekwb@gumed.edu.pl; 3 Department of Periodontology and Oral Mucosa Diseases, Medical University of Gdańsk, Dębowa 1a, 80-204 Gdansk, Poland; 4 Medicare Dental Clinic, Popieluszki 17a/102, 01-595 Warsaw, Poland; mkrasny@op.pl

**Keywords:** NMDA antagonists, memantine, Alzheimer’s disease, neural networks, computer simulation, virtual therapy, in silico trials

## Abstract

The variability in clinical trial results on memantine treatment of Alzheimer’s disease remains incompletely explained. The aim of this in silico study is a virtual memantine therapy for Alzheimer’s disease that provides a different perspective on clinical trials; An in silico randomised trial using virtual hippocampi to treat moderate to severe Alzheimer’s disease with doses of memantine 3–30 µM compared to placebo. The primary endpoint was the number of impulses (spikes). Secondary endpoints included interspike interval and frequency; The number of virtual moderate-AD hippocampal spikes was significantly lower, at 1648.7 (95% CI, 1344.5–1952.9), versus those treated with the 3 µM dose, 2324.7 (95% CI, 2045.9–2603.5), and the 10 µM dose, 3607.0 (95% CI, 3137.6–4076.4). In contrast, the number of virtual spikes (spikes) of severe AD of the hippocampus was significantly lower, at 1461.8 (95% CI, 1196.2–1727.4), versus those treated with the 10 µM dose, at 2734.5 (95% CI, 2369.8–3099.2), and the 30 µM dose, at 3748.9 (95% CI, 3219.8–4278.0). The results of the analysis of secondary endpoints, interspike intervals and frequencies changed statistically significantly relative to the placebo; The results of the in silico study confirm that memantine monotherapy is effective in the treatment of moderate to severe Alzheimer’s disease, as assessed by various neuronal parameters.

## 1. Introduction

Dementia is a very serious health problem affecting not only patients but also burdening families and ageing societies worldwide. Currently, approximately 47 million people worldwide experience dementia, and projections for the next 10 years indicate that it will affect nearly 80 million people. Of these cases, Alzheimer’s disease (AD) is the most common cause [1].

Alzheimer’s disease is a neurodegenerative disorder characterised by a progressive loss of cognitive function, memory, difficulty in understanding spatial relationships and problems in performing everyday tasks [2,3]. New therapeutic strategies, with particular emphasis on methods closely related to the pathogenetic background of AD, which, by limiting the accumulation of amyloid β (Aβ) and tau protein, may modify the natural course of the disease [4,5].

Memantine, which received European marketing approval in 2002 and US Food and Drug Administration (FDA) approval in 2003, is mainly used to treat moderate to severe forms of AD [6]. The results of several clinical trials show that memantine exerts neuroprotective effects [7,8,9]. Memantine shows beneficial effects in conditions of excessive glutamatergic neurotransmission by inhibiting toxicity [10,11,12]. Studies show that memantine, especially in terms of its effects on functioning, appears to be more effective in patients with more advanced stages of dementia [13], but its effects on cognitive function are also observed in all stages of dementia [7,8,14]. The results of a meta-analysis comparing the efficacy of different treatment regimens and placebo in AD show that the combination of memantine and donepezil had a better effect on cognitive function, among other things, but was less tolerated by patients compared to memantine or placebo alone [15]. The results of another meta-analysis show that galantamine and rivastigmine, like memantine, can delay cognitive impairment in patients with mild to severe AD [16].

Computer models can be used in many different ways to reduce, refine and partly replace animal and human experiments [17,18]. The efficacy of a drug and its value in clinical practice are assessed during phase III trials using randomisation and a control group. The benefits of in silico clinical trials are a reduction in human trials, possible replacement of, e.g., phase III trials, confirmation of model predictions as a way to increase confidence before investing in a trial, lower development cost and/or shorter time to market for new medical products [19]. Memantine has the best pharmacological profile and tolerability according to preclinical investigations and clinical trials using noncompetitive NMDA receptor antagonists [6,20,21,22].

Because of the limitations of modern research methods, we cannot examine the nervous system in natural conditions. Computer models of neurons [23] and neural networks [24,25] are two methods for studying the nervous system’s functioning. Understanding the process of neurodegeneration in Alzheimer’s disease is aided by computer models of synaptic degradation in the hippocampus for various stages of synaptic loss [26]. Other simulation experiments, on the other hand, demonstrate that generating gamma oscillations in the hippocampus can help with the pathophysiology of Alzheimer’s disease [27]. Artificial neural networks have been successfully applied in nuclear medicine and in the detection of Alzheimer’s disease based on cerebral perfusion single-photon emission computed tomography (SPECT) data [28,29]. A computer simulation environment of the *N*-methyl-d-aspartate receptor encompassing biological principles of channel activation by high extracellular glutamic acid concentration was used in three models of excitotoxicity severity [30,31].

In our study, we are undertaking an in silico clinical trial with a randomised virtual hippocampus, testing the efficacy of a drug in moderate to severe dementia in Alzheimer’s disease. Our simulation study opens up remarkable new scenarios in which a medical product, drug or device, can be developed and tested for in silico efficacy.

## 2. Results

### 2.1. Number of Spikes in CA1, CA3 and DG

**Figure 1:**
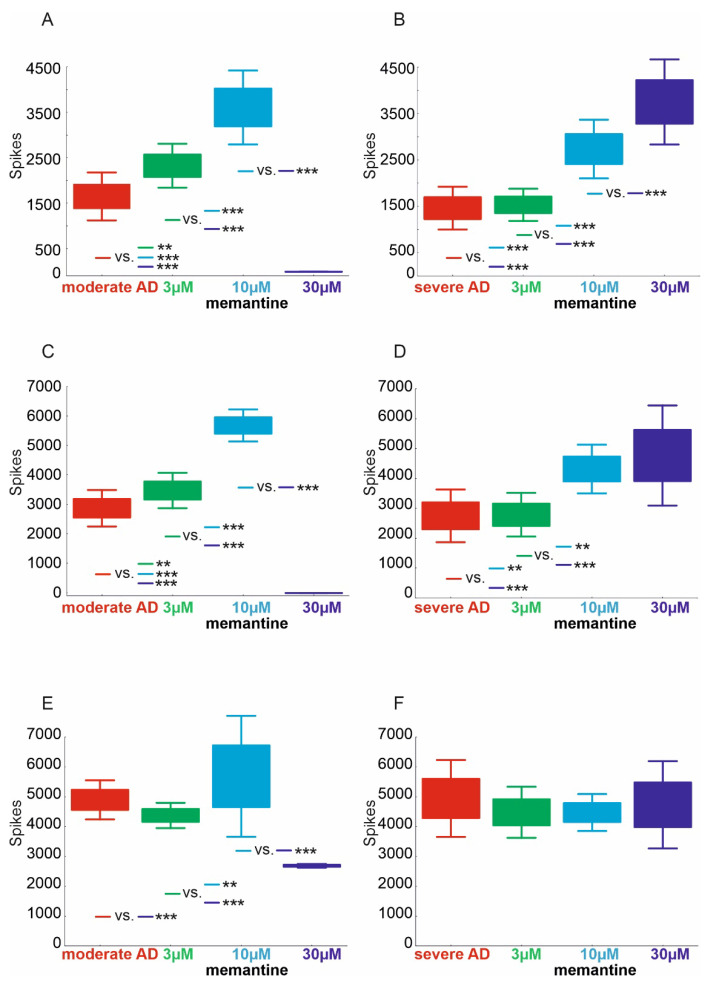
Comparison of number of spikes in virtual therapy of memantine treatment at three concentrations: 3 µM, 10 µM and 30 µM. (**A**) CA1 region moderate AD, (**B**) CA1 region severe AD, (**C**) CA3 region moderate AD, (**D**) CA3 region severe AD, (**E**) DG region moderate AD, (**F**) DG region severe AD (** *p* < 0.01, *** *p* < 0.001).

The mean number of spikes of area CA1 in the virtual moderate-AD hippocampal group treated with memantine at doses of 3–30 µM was statistically significantly different (*p* < 0.000001). The number of spikes of virtual hippocampal AD was significantly lower, at 1648.7 (95% CI, 1344.5–1952.9), compared to the number of spikes of virtual hippocampal AD treated with the 3 µM dose, 2324.7 (95% CI, 2045.9–2603.5), and the 10 µM dose, 3607.0 (95% CI, 3137.6–4076.4). Furthermore, the number of virtual AD spikes of the hippocampus treated with the 3 µM dose was significantly lower compared to the number of spikes treated with the 10 µM and 30 µM doses. In contrast, the number of spikes was significantly higher in the group treated with the 10 µM dose relative to those treated with the 30 µM dose (Figure 1A).

The mean number of CA1 area spikes in the virtual severe hippocampal AD group treated with memantine at doses of 3–30 µM was statistically significantly different (*p* < 0.000001). The number of spikes of virtual hippocampal AD was significantly lower, 1461.8 (95% CI, 1196.2–1727.4), compared to the number of spikes of virtual hippocampal AD treated with the 10 µM dose, 2734.5 (95% CI, 2369.8–3099.2), and the 30 µM dose, 3748.9 (95% CI, 3219.8–4278.0). There were no statistically significant differences in the number of hippocampal virtual AD spikes against those treated with memantine at a dose of 3 µM. In addition, the number of spikes of virtual hippocampal ADs treated with the 3 µM dose was significantly lower, 1533.0 (95% CI, 1333.1–1732.9), versus those treated with the 10 µM and 30 µM doses. The virtual AD group of hippocampi treated with memantine at a dose of 10 µM had a significantly higher number of spikes compared to those treated with a dose of 30 µM (Figure 1B).

The mean number of spikes of area CA3 in the virtual moderate-AD hippocampal group treated with memantine at doses of 3–30 µM was statistically significantly different (*p* < 0.000001). The number of spikes of virtual hippocampal AD was significantly lower, 2863.4 (95% CI, 2505.4–3221.4), versus the number of spikes of virtual hippocampal AD treated with a 3 µM dose, 3465.7 (95% CI, 3118.7–3812.7), and 10 µM dose, 5676.5 (95% CI, 5360.7–5992.3). However, the number of spikes treated with the 30 µM dose was significantly lower, 0.4 (95% CI, −0.5–1.3), compared to the moderate-AD group. In contrast, the 10 µM treatment group had a significantly higher number of spikes compared to the 30 µM treatment group (Figure 1C).

The mean number of spikes of area CA3 in the virtual severe-AD hippocampal group treated with memantine at doses of 3–30 µM was statistically significantly different (*p* < 0.000001). The number of spikes of virtual hippocampal AD was significantly lower, 2747.3 (95% CI, 2237.5–3257.1), compared to the number of spikes of virtual hippocampal AD treated with the 10 µM dose, 4316.6 (95% CI, 3846.1–4787.1), and the 30 µM dose, 4769.1 (95% CI, 3800.7–5737.5). There were no statistically significant differences in the number of hippocampal virtual AD spikes against those treated with memantine at a dose of 3 µM. In addition, the number of spikes of virtual hippocampal ADs treated with the 3 µM dose was significantly lower, 2785.1 (95% CI, 2361.9–3208.3), versus those treated with the 10 µM and 30 µM doses. The group of virtual hippocampal ADs treated with memantine at a dose of 10 µM showed no significant differences in the number of spikes compared to those treated with a dose of 30 µM (Figure 1D).

The mean number of DG area spikes in the virtual moderate-AD hippocampal group treated with memantine at doses of 3–30 µM was statistically significantly different (*p* < 0.000001). The number of spikes of virtual hippocampal ADs was significantly higher, 4896.1 (95% CI, 4517.3–5274.9), versus the number of spikes of virtual hippocampal ADs treated with the 30 µM dose, 2686.5 (95% CI, 2650.8–2722.2). In contrast, the number of spikes treated with a dose of 3 µM was significantly lower, 4375.3 (95% CI, 4130.7–4619.9), compared to the group treated with a dose of 10 µM, 5886.2 (95% CI, 4716.7–7055.7). In contrast, the number of pulses was significantly lower in the 30 µM-dose-treated group compared to the 3 µM- and 10 µM-dose-treated groups (Figure 1E). No statistically significant differences were found for the other comparisons. The mean number of spikes of the DG area in the virtual severe-AD hippocampal group treated with memantine at doses of 3–30 µM was not statistically significantly different (*p* = 0.6051) (Figure 1F).

### 2.2. Interspike Interval and CA1 Frequency

**Figure 2:**
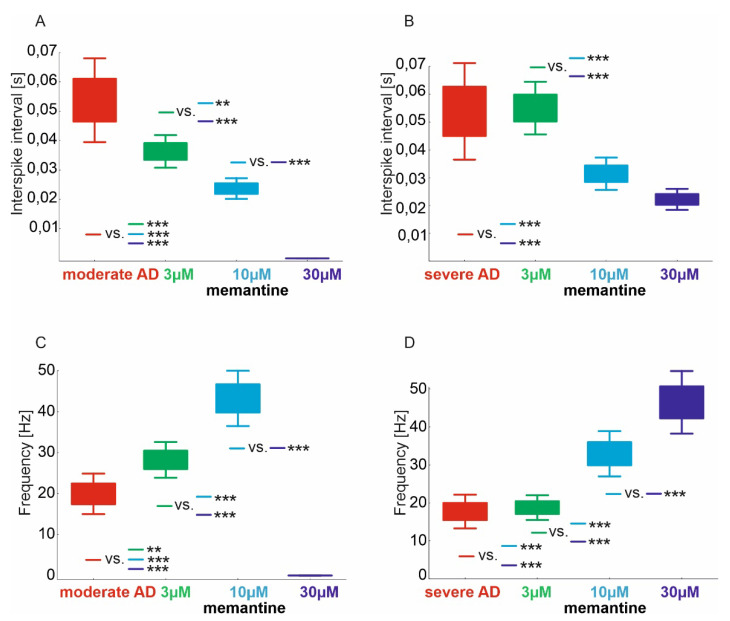
Comparison of ISI and frequency in virtual therapy of memantine treatment at three concentrations: 3 µM, 10 µM and 30 µM of CA1 region. (**A**) ISI of moderate AD, (**B**) ISI of severe AD, (**C**) frequency of moderate AD, (**D**) frequency of severe AD (** *p* < 0.01, *** *p* < 0.001).

The mean ISI of area CA1 in the hippocampal virtual moderate-AD group treated with memantine at doses of 3–30 µM was statistically significantly different (*p* < 0.000001). The moderate-AD group had a significantly higher ISI of 0.054 s (95% CI, 0.043–0.065) compared with those treated with the memantine 3 µM dose of 0.036 s (95% CI, 0.032–0.041), the 10 µM dose of 0.024 s (95% CI, 0.021–0.026) and the 30 µM dose of <0.001 (95% CI, not evaluable). Similar results were obtained in the 3 µM-dose-treated group, where the ISI was significantly higher compared with the 10 µM- and 30 µM-dose-treated groups and in the 10 µM- versus 30 µM-dose-treated group (Figure 2A).

The mean ISI of area CA1 in the virtual severe-AD hippocampal group treated with memantine at doses of 3–30 µM was statistically significantly different (*p* < 0.000001). In the severe-AD group, the ISI was significantly higher, at 0.061 s (95% CI, 0.048–0.074), compared with those treated with the 10 µM memantine dose, at 0.031 s (95% CI, 0.027–0.036), and the 30 µM dose, at 0.022 s (95% CI, 0.019–0.025). The same was obtained in the group treated with the 3 µM dose, where the ISI was significantly higher at 0.055 s (95% CI, 0.048–0.062) compared with those treated with the 10 µM and 30 µM doses (Figure 2B).

The mean value of the CA1 area pulse frequency in the hippocampal virtual moderate-AD group treated with memantine doses of 3–30 µM was statistically significantly different (*p* < 0.000001). In the moderate-AD group, the frequency was significantly lower at 19.9 Hz (95% CI, 16.1–23.7) versus those treated with the 3 µM memantine dose at 28.2 Hz (95% CI, 24.9–31.6) and the 10 µM dose at 43.2 Hz (95% CI, 38.0–48.4). However, against those treated with the 30 µM dose, CA1 pulse frequency <0.1 (95% CI, not possible to assess) was significantly higher. A similar result was obtained in the group treated with the 3 µM dose, where the frequency was significantly lower against those treated with the 10 µM dose, but significantly higher against those treated with the 30 µM dose. The frequency of pulses treated with the 10 µM dose was significantly higher compared to those treated with the 30 µM dose (Figure 2C).

The mean value of the pulse frequency from area CA1 in the virtual severe hippocampal AD group treated with memantine at doses of 3–30 µM was statistically significantly different (*p* < 0.000001). In the severe-AD group, the frequency was significantly lower at 17.7 Hz (95% CI, 14.3–21.1) compared with subjects treated with memantine at 10 µM at 32.9 Hz (95% CI, 28.4–37.5) and at 30 µM at 46.5 Hz (95% CI, 40.1–52.8). The same was obtained in the group treated with the 3 µM dose, where the frequency was significantly lower at 18.7 Hz (95% CI, 16.2–21.2) compared to those treated with the 10 µM and 30 µM doses. The frequency of the pulses treated with the 10 µM dose was significantly higher compared to those treated with the 30 µM dose (Figure 2D).

### 2.3. Assessment of Relations of the Number of Spikes CA1, CA3 and DG Regions

**Figure 3:**
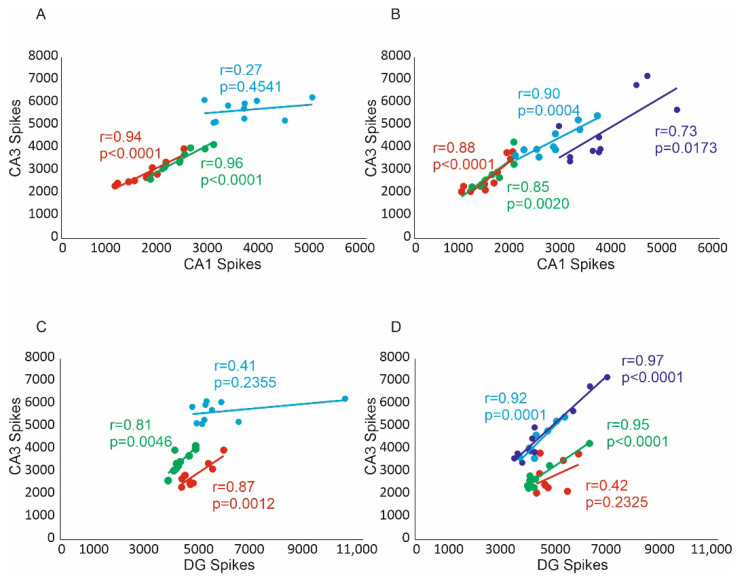
The correlation analysis of the number of spikes and CA1, CA3 and DG regions in virtual therapy of memantine treatment at three concentrations: 3 µM, 10 µM and 30 µM of CA1 region (red—AD, green—3 µM, blue—10 µM and violet—30 µM). Information in the hippocampus flows in the direction of DG → CA3 → CA1. The flow of information in CA3 → CA1 (**A**,**B**) and DG → CA3 (**C**,**D**). Relationships between number of spikes CA3, CA1 regions and DG, CA3 regions. (**A**) CA1 and CA3 of moderate AD, (**B**) CA1 and CA3 of severe AD, (**C**) DG and CA3 of moderate AD, (**D**) DG and CA3 of severe AD.

Positive and statistically significant correlations were found for the number of spikes of CA1 and CA3 in the group of virtual hippocampal AD moderates (correlation coefficient r = 0.94, *p* < 0.0001) and those treated with the 3 µM dose (correlation coefficient r = 0.96, *p* < 0.0001). In contrast, there was no statistically significant relationship in the virtual moderate-AD hippocampal group treated with the 10 µM dose (correlation coefficient r = 0.27, *p* = 0.4541) (Figure 3A).

Correlation analysis showed positive and statistically significant correlations of the number of spikes of CA1 and CA3 areas in the virtual severe-AD hippocampal group (correlation coefficient r = 0.88, *p* < 0.0001), treated with a dose of 3 µM (correlation coefficient r = 0.85, *p* = 0.0020), a dose of 10 µM (correlation coefficient r = 0.90, *p* = 0.0004) and 30 µM (correlation coefficient r = 0.73, *p* = 0.0173) (Figure 3B).

Positive and statistically significant correlations were found for the number of DG and CA3 area spikes in the hippocampal virtual moderate-AD group (correlation coefficient r = 0.87, *p* = 0.0012) and those treated with the 3 µM dose (correlation coefficient r = 0.81, *p* = 0.0046). In contrast, there was no statistically significant relationship in the virtual moderate-AD hippocampal group treated with the 10 µM dose (correlation coefficient r = 0.41, *p* = 0.2355) (Figure 3C).

Correlation analysis showed positive and statistically significant correlations of the number of DG and CA3 area spikes in the virtual severe-AD hippocampal group treated with the 3 µM dose (correlation coefficient r = 0.95, *p* < 0.0001), treated with the 10 µM dose (correlation coefficient r = 0.92, *p* = 0.0001) and the 30 µM dose (correlation coefficient r = 0.97, *p* < 0.0001). In contrast, there was no statistically significant relationship in the hippocampal virtual severe-AD group (correlation coefficient r = 0.42, *p* = 0.2325) (Figure 3D).

## 3. Discussion

Clinical trials testing monotherapy in Alzheimer’s disease have been conducted in the USA, Japan, Austria, the UK, China, and multiple other countries, including a dozen European countries [32,33,34,35,36,37,38,39,40]. In clinical trials, the primary endpoint is cognitive function as measured by SIB, ADAS, SMMSE [41]. Five studies showed no statistically significant differences in cognitive function versus placebo [33,34,35,37,39]. The mean cognitive function scores of patients with AD and the control group were −4.10 to 2.41 vs. −2.80 to 5.60, respectively. In four studies, memantine monotherapy improved cognitive function scores in the group of patients with AD relative to placebo, from −0.80 to 4.00 vs. 1.10 to 10.10, respectively. The result of a meta-analysis of the aforementioned studies showed that memantine monotherapy was statistically significant and improved cognitive function. In addition, the results of the meta-analysis of the secondary endpoint showed that daily living activities, global function assessment scores and stage of dementia assessment scores were significantly improved.

In our in silico study, we obtained confirmation of the efficacy of memantine in moderate to severe dementia in the course of Alzheimer’s disease. The performed model together with the virtual therapy confirm the results obtained in clinical trials. In areas CA3-CA1, we obtained a statistically significant improvement in the number of spikes relative to moderate AD with virtual therapy at doses of 3–10 µM. In contrast, in a model of severe AD we obtained an improvement of virtual therapy with doses of 3–30 µM. In studies that used animal models, it has been shown that exceeding a dose of 20 mg/kg can cause side effects [42]. Experiments suggest that memantine inhibits pathological alterations in the hippocampi [42], and that it prevents neuronal death in rats when given before NMDA injections [43]. In our study, we confirm that the use of memantine in models of excitotoxicity severity (moderate and severe) resulted in shortened ISI and increased number of spikes, or frequency. We obtained statistically significant differences in the number of spikes, interspike intervals and frequency when treated with memantine at doses of 3–30 µM.

Memantine’s favourable effects on cognitive function in Alzheimer’s patients have been proven in multiple multicentre studies involving individuals with moderate to severe Alzheimer’s disease who received memantine monotherapy [44]. Our simulation experiments confirm that the use of memantine in models of excitotoxicity with severity from moderate to severe, as measured by number of spikes, results in a statistically significant correlations with drug dose.

The results of clinical trials suggest that memantine monotherapy is effective in the treatment of AD when assessed using various scales. The available tools assessed cognitive function, behavioural impairment, activities of daily living, and stage of dementia, among others. Our simulation study also evaluates the efficacy of virtual memantine therapy based on various parameters, including spikes, frequency, and ISI.

## 4. Materials and Methods

### 4.1. Virtual Hippocampus of AD Population

Virtual hippocampi of AD patients with moderate to severe severity were included in the study. Inclusion criteria included the degree of synaptic decay and the severity of excitotoxicity. In the moderate and severe disease arm of the study, the respective synaptic decays in the hippocampus were defined for a phase of 9% and 18% synapse loss by modelling AD dynamics. The process of modelling the severity of AD involved disabling one-by-one the connections to entorhinal cortex layer 2 (EC2) and connections to EC2 on inhibitory interneurons of dentate gyrus (DG) granule cells and CA3 pyramidal neurons. The methodology for simulating the degree of synaptic decay describing AD disease dynamics was based on the formalism of previous studies [23,24,25,26,27,30,31]. Similarly, potentiation of excitotoxicity was modelled by increasing extracellular glutamate concentration and over-stimulation of NMDA receptors. Using the formalism from previous studies in moderate to severe disease, the parameter “powerA” was progressively changed in the following equation to model the phenomenological event:

power = powerA(M − ReP),(1)

where powerA = 9 is a parameter and M is the actual value of synaptic function SF(t) for excitatory postsynaptic potentials (ln(9) = 2.197 for control, ln(63) = 4.143 for moderate AD and ln(135) = 4.905 for severe AD).

### 4.2. Scheme of the Study

**Figure 4:**
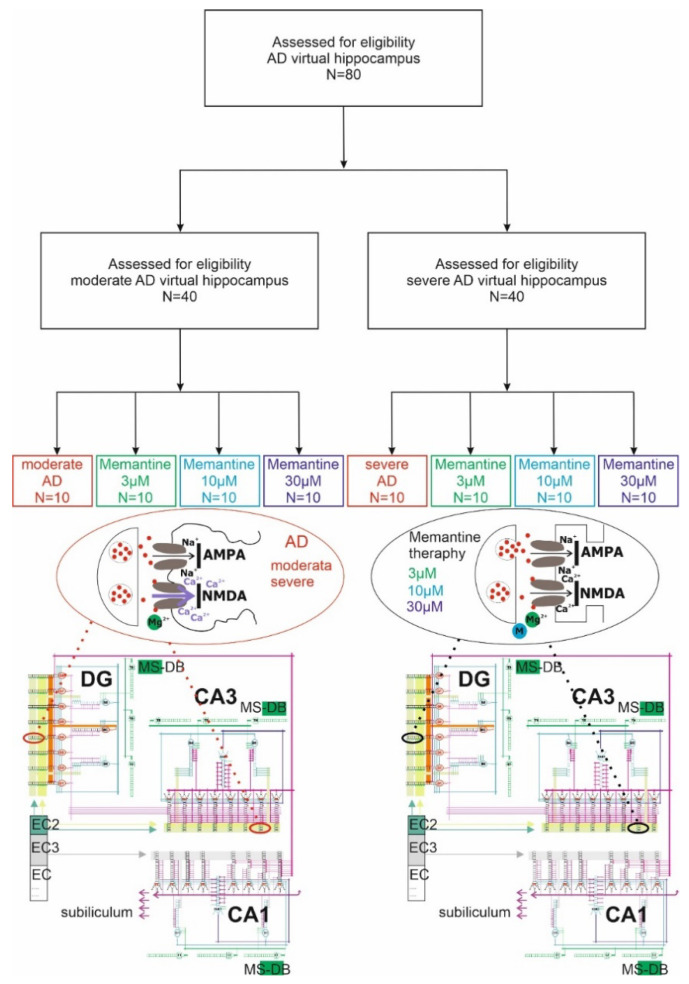
Simulation diagram of hippocampal network. Each group was randomised to one of four subgroups, where one was a control and the other three received virtual memantine therapy at doses of 3 µM (green), 10 µM (blue) and 30 µM (violet). Activation of the receptor caused by neurotoxic factors results in the release of Mg^2+^ and an uncontrolled influx of Ca^2+^ into the cell—red circle on the left. The depolarization caused by a strong stimulus is sufficient to remove the blockade of the memantine channel and to allow the influx of calcium ions into the cell—black circle on the right.

The study used 80 virtual AD models of the hippocampus, which were divided, according to the degree of synaptic decay and the severity of excitotoxicity, into two groups: moderate AD and severe AD, with 40 in each (Figure 4). Each group was randomised to one of four subgroups, where one was a control and the other three received virtual memantine therapy at doses of 3–30 µM. The final study design included 140 virtual simulations of hippocampal AD models, including 80 that matched AD pathology and 60 treated with memantine.

### 4.3. Virtual Memantine Therapy

Virtual memantine therapy of AD patients was performed using three concentrations: 3 µM, 10 µM and 30 µM. Modelling exploited the fact that memantine inhibits NMDA receptor currents in a concentration-dependent manner with IC50 values (concentration causing 50% inhibition) in the range 0.5–30 µM at hyperpolarised membrane potentials (−30 to −70 mV). The activation of NMDA receptors and the opening of ion channels depend on the synaptic membrane potential. During the period of resting membrane potential, magnesium ions (Mg^2+^) that are present in the extracellular space enter the channel, and by closing its lumen, temporarily inhibit the flow of calcium ions (Ca^2+^) and sodium ions (Na^+^). However, when at the same time there is a strong stimulation of postsynaptic receptors by glutamic acid and the value of the total potential is higher than the NMDA channel opening threshold for calcium ions (−68 mV), then the unblocked channel becomes permeable to Na^+^ and Ca^2+^ ions, which penetrate the cell and cause its stimulation. Using the ability to control the NMDA channel opening threshold in our model and the knowledge that memantine is a voltage-dependent NMDA receptor antagonist, a virtual treatment in the concentration range 3–30 µM was performed.

### 4.4. The Model of Hippocampus

**Figure 5:**
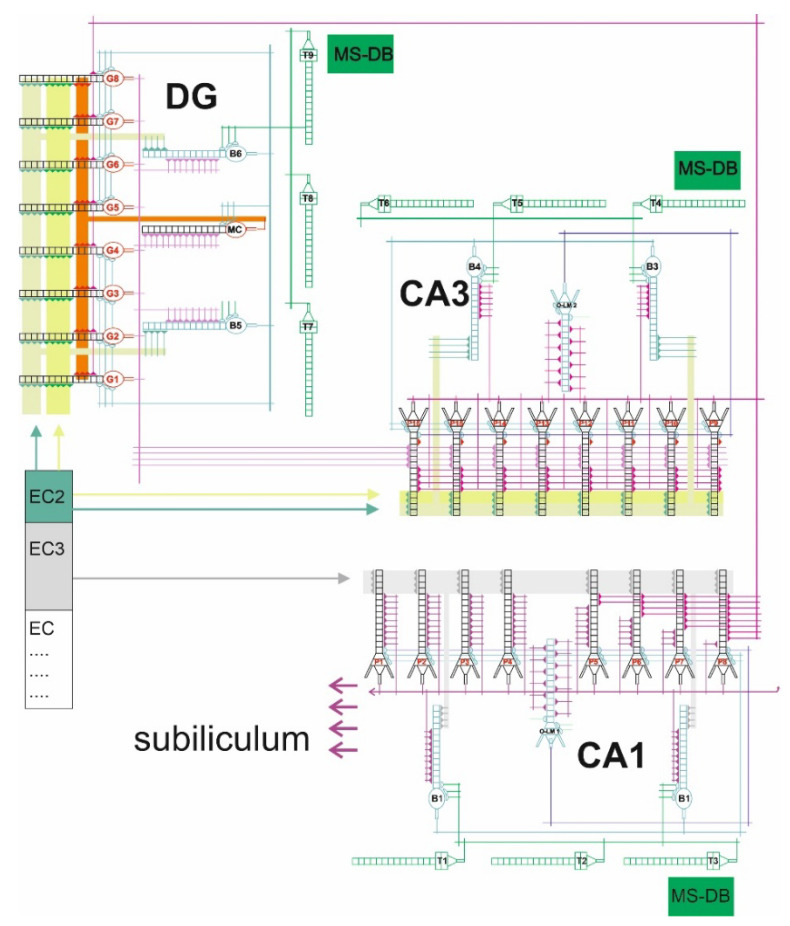
DG-CA3-CA1 hippocampal formation microcircuit, with the dentate gyrus (DG) region on the left, CA3 on the right and CA1 bottom. Major cell types and connectivity: (G1–G8)—granule cells, (P1–P16)—pyramidal cells, (B1–B6) basket cells, O-LM1 and O-LM2 cells, (MC) mossy cell, (T1–T9)—GABAergic cells in the medial-septum–diagonal band (MS-DB) which provides the disinhibitory inputs on hippocampal GABAergic interneurons at theta rhythm.

The DG-CA3-CA1 mathematical–computational model used the formalism from previous studies. What is new is the increased number of neurons, which is 33 in the current study (Figure 5).

The DG region was composed of eight granule cells and three interneurons, two basket cells and an O-ML cell. In contrast, the CA3 and CA1 regions each contained eight pyramidal cells and three interneurons, two basket cells and one O-LM cell. All cells were sixteen-compartmented. Each dendrite had both excitatory and inhibitory synapses. Using register methodology, excitatory synaptic inputs had two registers associated with the glutamine receptors AMPA and NMDA. In contrast, the inhibitory synaptic input contained one register associated with the GABA receptor. In our model, each CA3 pyramidal cell received inhibitory synapses from basket cells and O-LM cells. In contrast, CA3 pyramidal cells received excitatory inputs from the second layer of the entorhinal cortex and the dentate gyrus. Basket cells received excitatory inputs from distal dendrites of the second layer of the entorhinal cortex and from neurons of the dentate. Inputs from EC2 and EC3 were phase-shifted relative to each other so that strong excitation from one was matched by weak excitation from the other. Each O-LM cell had excitatory inputs from CA3 pyramidal cells and inhibitory inputs from the septum, and the sources of inputs to CA1 were fibres from the third layer of the entorhinal cortex and Schaffer collaterals from the CA3 sector. In addition, our model simulated theta oscillation arriving through the septal–hippocampal pathway running through the vault and described by frequencies in the 4 Hz to 12 Hz band, temporally anchored in faster gamma oscillations [23,24,25,26,27,30,31].

### 4.5. The Assessed Parameters

For all simulations of virtual AD models of the hippocampus, the number of spikes was measured in three areas of the hippocampus: DG, CA3 and CA1. The interspike interval (time between subsequent action potentials) and frequency of action-potential generation in area CA1 were also assessed. Information flow in the hippocampus is from DG to CA3 and from CA3 to CA1, which receives processed information from both the medial (MEC) and lateral (LEC) entorhinal cortex via CA3 input.

### 4.6. Statistical Analysis

The description of computer simulation parameters used arithmetic means together with 95% confidence intervals. Graphical presentation in the form of box and whiskers plots used mean values, the box was the area mean ± standard error and the whiskers were the 95% CI for the mean. Shapiro–Wilk and Leven’s (Brown–Forsythe) tests of concordance were used to check for population origin with normal distribution and homogeneity of variance, respectively. The results of virtual memantine treatment were assessed by ANOVA, using Tukey’s post hoc test for statistical significance. Pearson correlation analysis was used to assess the degree and strength of association. Statistical analysis was performed using TIBCO Software Inc. (Palo Alto, CA, USA) (2017). Statistica (data analysis software system), version 13. http://statistica.io (accessed on 1 January 2020). A significance level of α = 0.05 was adopted.

## 5. Conclusions

The results of this in silico study confirm that memantine monotherapy is effective in the treatment of moderate to severe Alzheimer’s disease, as assessed by various neuronal parameters.

The most important limitation of in silico research is the realisation of what a mathematical and computer model can do. The predictions and results of the model are always a consequence of the knowledge we used to build such a model.

## Data Availability

Data is contained within the article.
